# Cost-Effectiveness of Antiretroviral Therapy for Multidrug-Resistant HIV: Past, Present, and Future

**DOI:** 10.1155/2012/595762

**Published:** 2012-11-08

**Authors:** Marianne Harris, Bohdan Nosyk, Richard Harrigan, Viviane Dias Lima, Calvin Cohen, Julio Montaner

**Affiliations:** ^1^AIDS Research Program, Providence Health Care Institute, B518 1081 Burrard Street, Vancouver, BC, Canada V6Z 1Y6; ^2^Department of Family Practice, Faculty of Medicine, University of British Columbia, Vancouver, BC, Canada V6T 1Z3; ^3^British Columbia Centre for Excellence in HIV/AIDS, Vancouver, BC, Canada V6Z 1Y6; ^4^Division of AIDS, Faculty of Medicine, University of British Columbia, Vancouver, BC, Canada V6T 1Z3; ^5^Community Research Initiative of New England, Boston, MA 02111, USA

## Abstract

In the early years of the highly active antiretroviral therapy (HAART) era, HIV with resistance to two or more agents in different antiretroviral classes posed a significant clinical challenge. Multidrug-resistant (MDR) HIV was an important cause of treatment failure, morbidity, and mortality. Treatment options at the time were limited; multiple drug regimens with or without enfuvirtide were used with some success but proved to be difficult to sustain for reasons of tolerability, toxicity, and cost. Starting in 2006, data began to emerge supporting the use of new drugs from the original antiretroviral classes (tipranavir, darunavir, and etravirine) and drugs from new classes (raltegravir and maraviroc) for the treatment of MDR HIV. Their availability has enabled patients with MDR HIV to achieve full and durable viral suppression with more compact and cost-effective regimens including at least two and often three fully active agents. The emergence of drug-resistant HIV is expected to continue to become less frequent in the future, driven by improvements in the convenience, tolerability, efficacy, and durability of first-line HAART regimens. To continue this trend, the optimal rollout of HAART in both rich and resource-limited settings will require careful planning and strategic use of antiretroviral drugs and monitoring technologies.

## 1. Introduction

In the early years of the highly active antiretroviral therapy (HAART) era starting in 1996, HIV with resistance to two or more agents in different antiretroviral classes posed a significant clinical challenge. Multidrug-resistant (MDR) HIV was an important cause of treatment failure and consequent morbidity and mortality [1]. In 1998, a large drug resistance survey among viremic HIV patients in the United States showed that 13% harbored three-class-resistant virus and 48% had two-class resistance [2]. With improvements in understanding of viral dynamics and the efficacy of first-line regimens, MDR HIV has become less common but has not disappeared entirely, as demonstrated in a Canadian cohort of HAART-treated individuals followed until 2007 [3]. While three-class antiretroviral drug resistance is now very unusual (2%), two-class resistance was observed in 17% of the cohort. Fortunately, treatment options for patients with MDR HIV have improved substantially in terms of effectiveness, toxicity, and tolerability, while remaining cost-effective in most cases.

## 2. Past (1996–2005) 

### 2.1. The HAART Era

The HAART era began in 1996, with the availability of triple drug regimens and clinical trial data demonstrating their efficacy [4, 5]. Around the same time, the availability of viral load testing improved both the understanding of viral dynamics in response to treatment and the ability to closely monitor treatment efficacy. The consequences of exposure to sequential drug regimens in the absence of full viral suppression were not fully appreciated until the advent of widespread HIV drug resistance testing around 2000. Most of the HIV-infected patients who had previously received less effective single and dual drug regimens had already developed drug-resistant HIV by this time. Furthermore, some early triple therapy regimens were less than optimally effective, due to relatively low potency of individual drugs and adherence challenges related to complex dosing with numerous pills and poor tolerability. As a result, during this time a significant proportion of treatment-exposed HIV-infected patients harbored MDR strains [2].

### 2.2. Treatment Strategies for Multidrug-Resistant HIV

In the first decade of HAART, treatment options for MDR HIV were very limited. Before 2003, all available antiretroviral agents belonged to one of the original three drug classes and considerable cross-resistance existed within each class. Given the limited effective drug options available at the time, various strategies were tried. Regimens including two protease inhibitors (PIs), saquinavir and ritonavir both in therapeutic doses, achieved good results in treatment-experienced patients who had not previously been exposed to PIs [6]; however, results of this dual PI-based regimen were variable in patients who had experienced indinavir or nelfinavir failure previously [7, 8]. Another strategy was to promote reemergence of drug-susceptible virus using structured treatment interruptions and thereby to enhance virologic response to subsequent antiretroviral therapy [9]. This strategy was abandoned when it was proven to be ineffective in promoting sustained virologic suppression or disease control and, more alarmingly, was associated with protracted CD4 declines [10]. A more successful treatment strategy was the use of multiple drug rescue therapy, also called mega-HAART or giga-HAART, whereby patients were treated with as many partially active agents as possible, generally six to eight [11–13]. This strategy proved to be effective for at least some of the patients, but adherence was a significant challenge because of regimen complexity and poor tolerability. Long-term sustainability was limited by toxicity and cost issues. 

### 2.3. Enfuvirtide

In 2003, 24-week results of two large randomized controlled studies (T-20 versus optimized background regimen only (TORO) 1 and 2) were published that demonstrated the efficacy of enfuvirtide, an HIV fusion inhibitor, for the treatment of patients with drug-resistant virus [14, 15] (Table 1). In the combined TORO 1 and 2 studies, the 48-week rates of virologic suppression to <50 copies/mL were 18.3% for enfuvirtide plus an optimized background regimen versus 7.8% for the optimized background regimen alone [16]. The drug received regulatory approval in Canada, the United States (US), and Europe in the same year (Table 2). Enfuvirtide represented an important breakthrough in that it was the first approved antiretroviral agent belonging to a new drug class, and hence cross-resistance with previous agents was not a problem. By the middle of the decade, enfuvirtide was considered a cornerstone of treatment for patients harboring MDR virus [17]. 

However, the complexity of synthesis and limited supply led to pricing of enfuvirtide in the US and Europe at $18,500 per person per year (calculated in 2001 US dollars, from $20,000 dollars in 2003 using the medical care component of the consumer price index), which was nearly twice as costly as any of the other approved single agents for treatment of HIV in use at the time [18–22]. In addition, enfuvirtide had to be used in combination with multiple other active antiretroviral agents [16, 23]. As a result, the annual cost of a combination antiretroviral regimen containing enfuvirtide was typically between $35,000 and $43,000 per person per year [18, 24]. 

Extrapolating from the 24- and 48-week TORO results, the cost-effectiveness of enfuvirtide in combination antiretroviral regimens was evaluated by Sax et al. in 2005 [25] and by Hornberger et al. in 2006 [26]. Using different methods of analysis, these two papers estimated the incremental cost-effectiveness ratio of enfuvirtide plus an optimized background regimen compared with an optimized background regimen alone to be $69,500 and $24,604, respectively, per quality-adjusted life-year (QALY) gained. (To calculate the incremental cost-effectiveness of an intervention, analyses must consider the added efficacy (generally measured in quality-adjusted life years (QALY)) and the additional costs of the intervention. The cost-effectiveness ratio is then calculated with incremental costs in the numerator and incremental benefits in the denominator ($/QALY). An intervention may be considered cost-effective if the additional benefit provided by the treatment is considered “worth" the additional cost. The World Health Organization (WHO) Commission on Macroeconomics and Health suggested that interventions may be considered very cost-effective when the cost-effectiveness ratio ($/QALY) is less than 1 times the per-capita gross domestic product (GDP) for an individual country and cost-effective when the ratio is less than 3 times the per-capita GDP [27]. As a point of reference, the estimated GDP per capita in Canada in 2010 is CDN$39,057 [28].) 

The results of these studies suggested that enfuvirtide-based regimens could represent a cost-effective option for treating individuals with MDR HIV and advanced disease at the time. The projected survival benefit of enfuvirtide plus an optimized background regimen becomes more apparent with longer-term followup [26]; however, long-term sustainability of enfuvirtide therapy was hampered by the need for twice daily subcutaneous injections and bothersome injection site reactions [29, 30]. Because of these issues and the availability of newer, more convenient oral agents, enfuvirtide is no longer widely used; however, there is no doubt that this agent saved the lives of many MDR-HIV-infected patients who would otherwise not have survived until other more sustainable options became available.

## 3. Present (2006–2011)

Starting in 2006, results of a number of clinical trials involving new antiretroviral agents were published in rapid succession (Table 1). Taken together, these studies represented a significant step forward in the treatment of MDR HIV: tipranavir (RESIST) [31], darunavir (POWER) [32], etravirine (with darunavir in DUET) [33, 34], maraviroc (MOTIVATE) [35], and raltegravir (BENCHMRK) [36]. The regulatory approval of these drugs in Canada, the US, and Europe between 2005 and 2008 enabled prescribers to effectively treat MDR HIV with more compact regimens including at least two and often three fully active agents, with a remarkable increase in the efficacy rates (Table 2). Full and durable viral suppression once again became a realistic goal in the treatment of these patients.

### 3.1. Tipranavir

Tipranavir was the first of a new generation of ritonavir- (r-) boosted PIs with efficacy against HIV strains that had reduced susceptibility to older PIs, including strains with multiple PI resistance-associated mutations [31]. Using 48-week data from the RESIST studies, Hubben et al. [40] and Simpson et al. [41] demonstrated that regimens including tipranavir/r could provide longer-term benefits in terms of reductions in AIDS events and corresponding QALY gains and life years saved, as compared to regimens based on older ritonavir-boosted PIs. These analyses found similar cost-effectiveness ratios for tipranavir/r versus comparator PI/r of €42,500 [40] and $56,517 [41] per QALY gained. Excluding patients also treated with enfuvirtide reduced the incremental cost-effectiveness ratio to $46,147 per QALY [41]. However, the use of tipranavir was limited by important tolerability and toxicity issues, including relatively uncommon but potentially fatal hepatotoxicity and intracranial hemorrhage [42].

### 3.2. Darunavir

Darunavir, another ritonavir-boosted PI that was also developed to treat PI-resistant HIV, is effective and generally safe and well-tolerated. The phase IIb POWER (performance of TMC-114/r when evaluated in treatment-experienced patients with PI resistance) trials [32, 37, 38] and the phase III TITAN (TMC114/r in treatment-experienced patients naïve to lopinavir) trial [43] demonstrated the efficacy of darunavir/r 600/100 mg twice daily among treatment-experienced HIV-infected adults. Subjects in the comparator arms received single (74%) or dual (23%) boosted PIs (mainly lopinavir, saquinavir, and/or amprenavir/fosamprenavir) in POWER and lopinavir/r in TITAN. A recent systematic review summarized the results of a number of cost-utility analyses conducted alongside these trials and demonstrated that the use of darunavir/r in this setting was cost-effective and, in some cases, cost saving [44].

As a result of complex PI resistance profiles, during this period there was some clinical use of two or more ritonavir-boosted PIs in a regimen [45]. A study comparing single to dual unboosted PIs showed modest benefit from the addition of the second PI [46]. The paucity of antiretrovirals from new classes led to clinical use of such dual PI regimens as one way to attempt to reestablish virologic suppression especially in patients with PI resistance. Given the results of the POWER studies, the ability of boosted darunavir to be used in place of a dual boosted PI regimen was explored in two similar randomized controlled trials of immediate substitution of ritonavir-boosted PIs with darunavir/r versus deferred substitution after 24 weeks [47, 48]. Together these two pilot-sized studies randomized 48 subjects (24 to each arm) who had undetectable plasma HIV RNA (<50 copies/mL) while receiving regimens including dual or triple boosted PIs. All 45 subjects who completed 24 weeks on study (23 in the immediate switch arms and 22 in the deferred switch arms) had undetectable viral load at week 24. Median CD4 cell count changes from baseline to week 24 were similar in the two arms. At week 48, virologic suppression was maintained in all but two subjects, one in each treatment arm. In this context, darunavir/r was shown to be an effective, compact, and relatively safe and tolerable option, as well as being less costly than two or three concomitant ritonavir-boosted PIs.

### 3.3. Etravirine, Maraviroc, and Raltegravir

The DUET, MOTIVATE, and BENCHMRK trials evaluated the efficacy of etravirine (plus darunavir/r), maraviroc, and raltegravir respectively, versus placebo, each given with an optimized background regimen of nucleoside reverse transcriptase inhibitors, PIs, and/or enfuvirtide [33–36]. 

Etravirine, the first available next-generation nonnucleoside reverse transcriptase inhibitor, has been successfully used for the treatment of HIV with some degree of resistance to the first-generation nonnucleoside reverse transcriptase inhibitors delavirdine, nevirapine, and efavirenz. Its main use, as supported by data from the DUET study, is in regimens also including a ritonavir-boosted PI, specifically darunavir [33, 34]. In the combined 48-week results of the DUET 1 and 2 studies among subjects with nonnucleoside- and PI-resistant HIV, virologic suppression to <50 copies/mL was observed in 61% of the etravirine group versus 40% of those randomized to placebo (both combined with an optimized background regimen that included darunavir/ritonavir) [39]. The antiviral activity of etravirine is reduced in the presence of three or more specific resistance-associated mutations [49].

Maraviroc and raltegravir, a CCR5 receptor antagonist and an integrase inhibitor, respectively, were the first available new-class options that could be given orally. While being an attractive agent in terms of its effectiveness and tolerability, maraviroc is limited to use in the treatment of patients with CCR5-tropic virus, which is an issue in treatment-experienced patients [50]. In the pooled results of the MOTIVATE 1 and 2 studies, the 48-week rates of virologic suppression to <50 copies/mL for treatment-experienced subjects with R5-tropic virus were 43% with maraviroc once daily and 46% with maraviroc twice daily versus 17% with placebo (all with an optimized background regimen) [35]. Economic evaluations of the MOTIVATE 1 and 2 studies have compared maraviroc plus optimized background therapy to optimized background alone. The incremental cost-effectiveness ratios per QALY gained were €23,457 and US$42,429 in analyses conducted in Spain and Mexico, respectively [51, 52]. The incremental cost-effectiveness ratio was found to be somewhat lower (more favorable) when maraviroc was modeled in individuals whose HIV was susceptible to two or fewer components of the background regimen and higher (less favorable) in individuals with HIV susceptible to three or more regimen components [52, 53].

Use of the integrase inhibitor raltegravir is not restricted by tropism, and the drug is effective against virus resistant to other drug classes. Given orally twice daily, it is relatively safe and well-tolerated, with minimal toxicity and drug interactions [54]. In the combined BENCHMRK 1 and 2 study results, the 48-week rates of virologic suppression to <50 copies/mL for treatment-experienced subjects with drug-resistant HIV were 62% with raltegravir versus 33% with placebo (both combined with an optimized background regimen) [36]. A pair of studies from Spain and Switzerland used data from the BENCHMRK 1 and 2 trials to assess the long-term cost-effectiveness of raltegravir plus background therapy as compared to background therapy alone. Incremental cost-effectiveness ratios for three years of treatment were calculated to be €22,908 and 42,751 Swiss francs in the two studies, respectively, and increased with longer treatment durations [55, 56]. By 2009, many patients receiving enfuvirtide in rescue therapy regimens were switched to raltegravir [57–60], with a significant improvement in patient acceptability and cost. Given the inconvenience of twice daily injections and the availability of raltegravir and other oral agents effective against MDR virus, the clinical role of enfuvirtide diminished, and it is seldom used today.

When they became approved and available, these newer agents were somewhat more expensive than the previous antiretrovirals (except enfuvirtide). The DUET, MOTIVATE, and BENCHMRK trials were conducted in treatment-experienced patients, where complex and expensive drug combinations are typically required. The average annual per patient cost of antiretrovirals for the active plus optimized background regimen arm versus placebo plus optimized background regimen was US$ 47,324 versus 38,267 in the DUET Trials, US$ 46,633 versus 36,404 in MOTIVATE, and US$ 45,484 versus 34,585 in BENCHMRK. Of note, in the three trials, the highest treatment costs were from nucleoside analogues (29-30% of total costs) and enfuvirtide (22–25% of total costs) [61]. The ability to design an effective regimen for a patient with MDR virus using fewer drugs than the previous multiple drug rescue therapy regimens permitted more cost-effective therapy. In addition, the improved safety and tolerability of most of these newer agents resulted in lower overall health care costs for the treatment of these individuals. By 2008 it became possible to successfully treat patients with MDR virus with a regimen including three active new agents: ritonavir-boosted darunavir, etravirine, and raltegravir, with or without partially effective nucleosides [62, 63]. 

## 4. Future: 2012 and Beyond

Despite evidence of ongoing risk behavior in patients infected with drug-resistant HIV [64], the spectre of widespread transmission of multidrug-resistant HIV has not materialized. This is probably related at least in part to the reduced fitness of multiply-mutant strains [65]. As combination antiretroviral regimens have become more potent in suppressing viral replication and genotypic resistance testing prior to treatment has become standard of care, the majority of HIV drug resistance emerges in the setting of incomplete adherence. First-line HAART regimens are becoming more convenient, more forgiving to missed doses, and better tolerated, so the emergence of resistance continues to become less frequent. As well, the long-term durability of modern HAART regimens is increasing [66]. 

On the other hand, the significant flourishing of new antiretroviral drugs and new drug classes that has occurred over the past several years is unlikely to be duplicated in the future. Fewer new agents are being developed for treating HIV, and some of them (e.g., rilpivirine) are aimed specifically at first-line therapy of treatment-naïve patients [67]. Two new integrase inhibitors, elvitegravir and dolutegravir, may play a role in treatment of drug-resistant HIV [68, 69]; however, elvitegravir demonstrates significant cross-resistance with raltegravir and is therefore unlikely to be effective for patients who have failed a raltegravir-based regimen [70]. Given the paucity of new antiretroviral drugs in the pipeline, the agents that are currently available will need to continue to control HIV replication for many years. Furthermore, many regimens in current use include drugs with a low genetic barrier to resistance (e.g., efavirenz, raltegravir), meaning that resistant HIV mutants can emerge relatively quickly as a consequence of virologic failure [49, 71, 72]. Therefore, strategic use of the available drugs and cautious management of patients will be critical for successful HIV management in the future. Adherence assessment and counseling will need to be integrated into routine clinical visits prior to and throughout antiretroviral treatment, in order to avert the emergence of drug-resistant HIV. In addition, routine pretreatment genotypic testing to detect primary resistance, regular viral load monitoring, and early genotypic testing in cases of virologic failure will be particularly important to prevent accumulation of resistance-associated mutations.

The burden of resistance could increase if new agents are not made available or their introduction is staggered, resulting in suboptimal regimens or functional monotherapy. This issue is particularly relevant to developing countries, where options for second-line and salvage therapy are limited by scarcity of resources; the newer drugs that are effective against drug-resistant HIV are costly and often not available in generic formulations. Furthermore, in these resource-limited settings, access to viral load monitoring and resistance testing may be restricted, if available at all. The absence of these laboratory tools to monitor the ongoing effectiveness of antiretroviral therapy can lead to significant delays in the diagnosis and management of virologic failure, with devastating consequences [64, 73]. The inevitable emergence of HIV drug resistance in these settings is a particular concern at a time when it has been recognized that sustained full suppression of viral replication is crucial, both to optimize the individual benefit of HAART and to decrease HIV transmission [74–77]. The optimal rollout of HAART in both rich and resource-limited settings will require careful planning to ensure access to the best available antiretroviral regimens and to the appropriate technologies to monitor their use.

## Figures and Tables

**Table 1:** Publications of pivotal studies of drugs for multidrug-resistant HIV.

Drug	Abbreviated study title and duration	Study treatment (*N*) and comparator (*N*) arms	Journal and date of publication
	TORO 1 (24 wks)	ENF + 3–5 drug OBT (328) versus 3–5 drug OBT (167)	NEJM, May 2003 [14]
	TORO 2 (24 wks)	ENF + 3–5 drug OBT (335) versus 3–5 drug OBT (169)	NEJM, May 2003 [15]
Enfuvirtide (ENF)	TORO 1 and 2		
	48 wk efficacy	ENF + OBT (661) versus OBT (334)	JAIDS, December 2005 [16]
	48 wk safety	ENF + OBT (663) versus OBT (334)	JAIDS, December 2005 [29]

Tipranavir (TPV)	RESIST (48 wks)	TPV/r + OBT (746) versus CPI/r + OBT (737)	Lancet, August 2006 [31]

	POWER 1 (24 wks)	DRV/r in 1 of 4 doses + OBT (255) versus CPI/r + OBT (63)	AIDS, February 2007 [37]
Darunavir (DRV)	POWER 2 (24 wks)	DRV/r in 1 of 4 doses + OBT (225) versus CPI/r + OBT (53)	AIDS, March 2007 [38]
	POWER 1 and 2 (48 wks)	DRV/r 600/100 mg BID + OBT (110) versus CPI/r + OBT (120)	Lancet, April 2007 [32]

	DUET 1 (24 wks)	ETR + DRV/r + OBT (304) versus DRV/r + OBT (308)	Lancet, July 2007 [33]
Etravirine (ETR)	DUET 2 (24 wks)	ETR + DRV/r + OBT (295) versus DRV/r + OBT (296)	Lancet, July 2007 [34]
	DUET 1 and 2 (48 wks)	ETR + DRV/r + OBT (599) versus DRV/r + OBT (604)	AIDS, November 2009 [39]

Raltegravir (RAL)	BENCHMRK 1 and 2 (48 wks)	RAL + OBT (462) + OBT (237)	NEJM, July 2008 [36]

Maraviroc (MVC)	MOTIVATE 1 and 2 (48 wks)	MVC QD + OBT (414) versus MVC BID + OBT (426) versus OBT (209)	NEJM, October 2008 [35]

OBT: optimized background therapy; CPI: comparator protease inhibitor; r: ritonavir; QD: once daily; BID: twice daily; NEJM: The New England Journal of Medicine; JAIDS: Journal of Acquired Immune Deficiency Syndromes.

**Table 2:** HIV drug approval/authorization dates.

Drug	Canada (Health Canada)	US (FDA)	Europe (EMEA)
Enfuvirtide	July 14, 2003	March 13, 2003	May 5, 2003
Tipranavir	November 21, 2005	June 22, 2005	October 25, 2005
Darunavir	July 28, 2006	June 23, 2006	February 12, 2007
Maraviroc	September 21, 2007	August 6, 2007	September 18, 2007
Raltegravir	November 27, 2007	October 12, 2007	December 20, 2007
Etravirine	August 23, 2008	January 18, 2008	August 28, 2008

US (FDA): United States Food and Drug Administration; EMEA: European Medicines Agency.
